# Emerging Trends and Research Foci in Tumor Microenvironment of Pancreatic Cancer: A Bibliometric and Visualized Study

**DOI:** 10.3389/fonc.2022.810774

**Published:** 2022-04-19

**Authors:** Kaiwen Wu, Ye Liu, Lei Liu, Yunlan Peng, Honglin Pang, Xiaobin Sun, Demeng Xia

**Affiliations:** ^1^ Department of Gastroenterology, The Third People’s Hospital of Chengdu, The Affiliated Hospital of Southwest Jiaotong University, Chengdu, China; ^2^ Southwest Jiaotong University College of Medicine, Southwest Jiaotong University Affiliated Chengdu Third People’s Hospital, Chengdu, China; ^3^ Naval Medical University, Shanghai, China; ^4^ Medical Research Center, Third People’s Hospital of Chengdu, Affiliated Hospital of Southwest Jiaotong University, Chengdu, China; ^5^ Luodian Clinical Drug Research Center, Shanghai Baoshan Luodian Hospital, Shanghai University, Shanghai, China

**Keywords:** pancreatic cancer, tumor microenvironment, bibliometric study, hotspot, glutamine metabolism liposome

## Abstract

**Background:**

Pancreatic cancer (PC) is a serious disease with high mortality. The tumor microenvironment plays a key role in the occurrence and development of PC. The purpose of this study is to analyze trends by year, country, institution, journal, reference and keyword in publications on the PC microenvironment and to predict future research hotspots.

**Methods:**

The Web of Science Core Collection was used to search for publications. We analyzed the contributions of various countries/regions, institutes, and authors and identified research hotspots and promising future trends using the CiteSpace and VOSviewer programs. We also summarized relevant completed clinical trials.

**Results:**

A total of 2,155 papers on the PC microenvironment published between 2011 and 2021 were included in the study. The number of publications has increased every year. The average number of citations per article was 32.69. The USA had the most publications, followed by China, and a total of 50 influential articles were identified through co-citation analysis. Clustering analysis revealed two clusters of keywords: basic research and clinical application. The co-occurrence cluster analysis showed glutamine metabolism, carcinoma-associated fibroblasts, oxidative phosphorylation as the highly concerned research topics of basic research in recently. The three latest hot topics in clinical application are liposomes, endoscopic ultrasound and photodynamic therapy.

**Conclusion:**

The number of publications and research interest have generally increased, and the USA has made prominent contributions to the study of the tumor microenvironment of PC. The current research hotspots mainly focus on energy metabolism in the hypoxic tumor microenvironment, cancer associated fibroblasts in regulating the tumor microenvironment, accurate diagnosis, drug delivery and new treatments.

## Introduction

Pancreatic cancer (PC) is a common digestive system tumor and has become the seventh leading cause of cancer death (4.7%). It is predicted to surpass breast cancer as the third leading cause of cancer death by 2025 (1). Because of its insidious onset, PC is difficult to diagnose at the early stage. More than 80% of PCs are already in the advanced stage when detected, and the opportunity for surgical resection is often missed due to local infiltration and long-term metastasis (2). Thus, the prognosis is relatively poor, the pathogenesis and treatment strategies of PC have always been research hotspots. In recent years, researchers have found that as a cancer progresses, it will hijack the physiological response of the surrounding interstitium and use its immune, vascular, and connective tissue elements to create an environment conducive to tumor growth, namely, the tumor microenvironment (TME). The TME is composed of tumor cells, stromal cells, and extracellular components, such as interstitial tissues near tumor cells (3, 4). The TME provides conditions for the occurrence, development, invasion, and metastasis of PC (5). Institutions and researchers aiming to target the relationship between tumor cells and the TME continue to offer hope for the treatment of PC. One such strategy is immune checkpoint treatment. An integral function of the immune system is its ability to distinguish between the self and non-self (6). For this reason, the immune system relies on multiple “checkpoints”, that is, molecules on specific immune cells that need to be activated or inactivated to start the immune response (7). Tumor cells often use these checkpoints to avoid being detected and attacked by the immune system. Checkpoint treatment has been studied as a new mode of cancer treatment, but its efficacy in PC is not satisfactory, which may be associated with the complex TME of PC (8). Recently, due to increasing interest in the TME of PC, hundreds of academic articles have been published on this topic. Therefore, the publication trends of this research area urgently need to be summarized to serve as a reference for future studies.

At present, we have entered the era of big data for scientific research. In the face of massive scientific research data, how to efficiently identify important research results, especially to sort out the development context and trend of research, has become a concern of scientific researchers. In this context, the bibliometric method may be a way to solve these problems. Bibliometrics is a commonly used method to summarize trends in publications and analyze the literature in a certain field. This approach relies on research methods such as statistics to visually summarize research progress in a field, predict research hotspots, and further evaluate trends in the field through a citation network (9–11). In recent years, bibliometrics has also been applied in critical diseases (12, 13), orthopedic diseases (14), respiratory diseases (15) and other fields (16). For example, Chan (17) investigated research hotspots for artificial intelligence in the diagnosis of breast cancer, and Chen (18) et al. summarized research progress on recurrent glioma. Although some studies have analyzed the developmental trend of targeted therapies for locally advanced and metastatic PC by means of bibliometrics (19), no bibliometric studies or visualization analyses of the TME of PC have as yet been reported.

**Figure 1:**
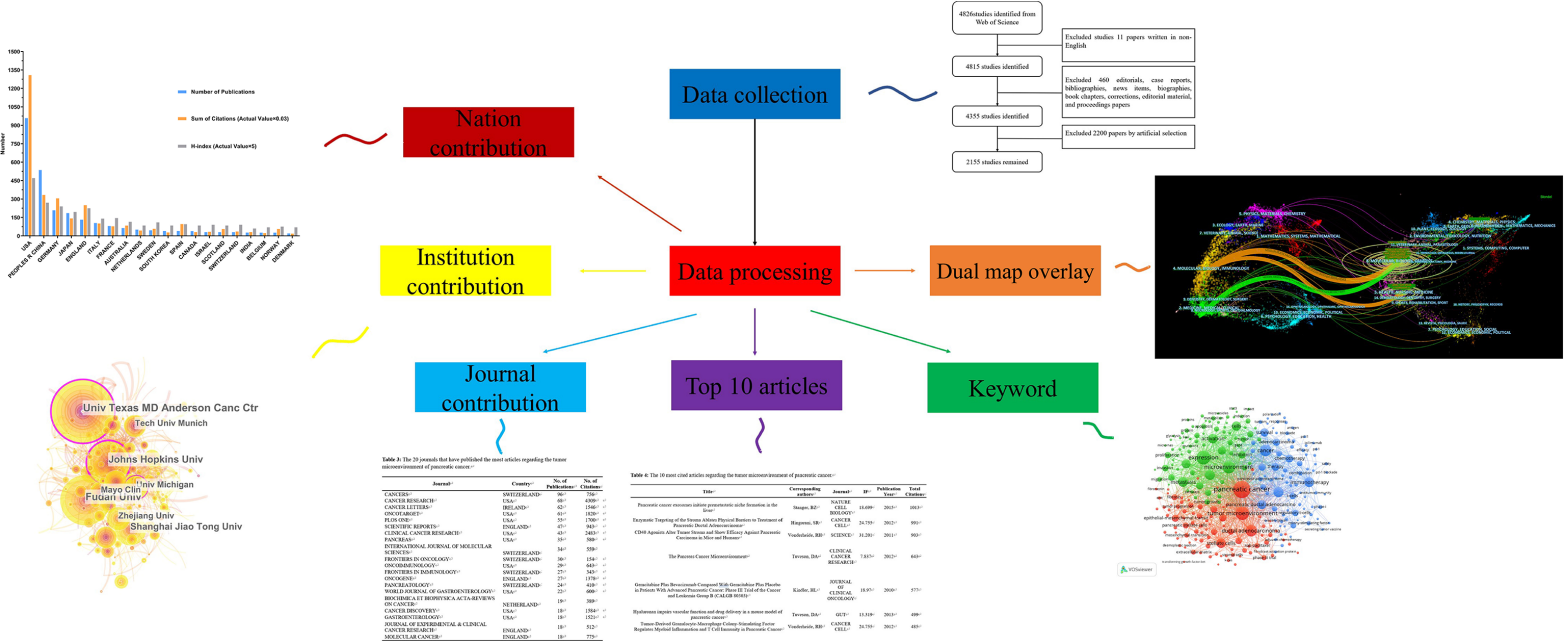
We searched the Web of Science Core Collection (WOSCC) on the TME of PC and performed a visualization analysis from the perspectives of countries, institutions, authors, journals, citations and keywords to predict future research hotspots in this field.

In the present study, we used bibliometric statistics to comprehensively analyze the literature related to the TME of PC by searching the Web of Science Core Collection (WOSCC). We performed a visualization analysis on the number of publications, citations, and research trends by country, author, and institution using CiteSpace, VOSviewer, and other software to predict future research hotspots in this field (**Figure 1**).

## Materials and Methods

### Data Sources and Search Strategies

Utilizing the WOSCC database, we conducted a comprehensive search of the literature on the TME of PC between 2011 and 2021. The search strategy was shown in the appendix. To prevent errors caused by database updates, all literature searches were completed on October 31, 2021. After excluding literature that did not meet the language and article type requirements, KWW and LL selected the rest of the literature, mainly by evaluating the title and abstract of articles to determine whether they should be included or excluded. For the uncertain literature, we downloaded the full text and conducted a more detailed evaluation. If there were questions, an experienced corresponding author (XBS) reviewed them. KWW and YL extracted titles, authors, keywords, abstracts, and references from all literature that met the criteria. All information was exported in.txt format. The raw data can be found in the **Supplemental File** (**Supplemental File 1**).

### Bibliometric Analysis

The WOSCC is commonly used in research on publications in the field of biomedicine. We used the WOSCC to describe the characteristics of the literature and analyzed the distribution of publications across various countries/regions, regions, institutions, authors, and journals. The RRI, H index, were extracted from WOSCC. Relative research interest (RRI) is based on the number of publications in a field in a given year among the number of publications in all fields in that year. The H-index is a bibliometric measure that combines quantity (publications) and impact (citations). It allows us to objectively characterize the scientific output of a researcher. The H-index may be superior to other commonly used bibliometric measures, such as the total number of papers published (Np) and the total number of citations garnered (Nc), as a representative measure of individual scientific achievement.

CiteSpace software (from 5.7. R2 64-bit, Chaomei Chen, Drexel University, USA) was used to analyze the screened literature, and a cocitation analysis was performed on authors, countries/regions, and institutions. Cluster and co-occurrence analyses were conducted on keywords, and from which the strongest citation bursts of the keywords were derived. The literature was then subjected to analysis by dual-map overlays. VOSviewer (a program operated by the Center for Science and Technology Studies at Leiden University that is used to create data maps) was used for the co-occurrence analysis of countries/regions, institutions, and authors. Cluster analysis was performed on the keywords.

We searched ClinicalTrials.gov (https://clinicaltrials.gov/) to summarize clinical studies on the TME of PC. The search strategy included the following: Condition or disease = Pancreatic Cancer; Other terms = Tumor Microenvironment; Study type = Interventional Study (Clinical Trials); Study Results = Study With Results, Status = Completed. A total of nine studies regarding the TME of PC were identified by manual screening of 11 records.

## Results

### Annual Publication Number and Trend

**Figure 2:**
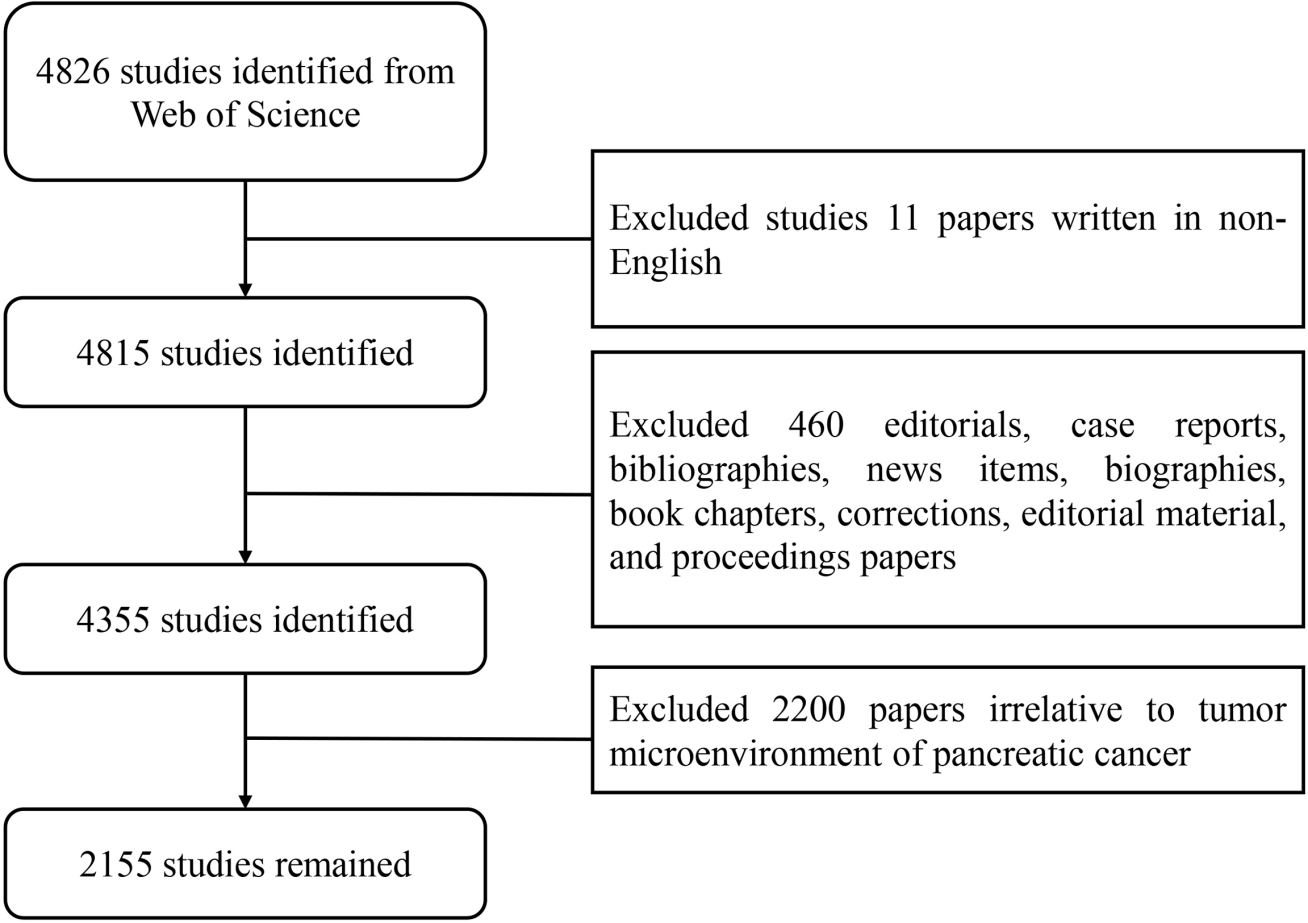
Flow chart of the screening process for research on the tumor microenvironment in pancreatic carcinoma.

**Figure 3:**
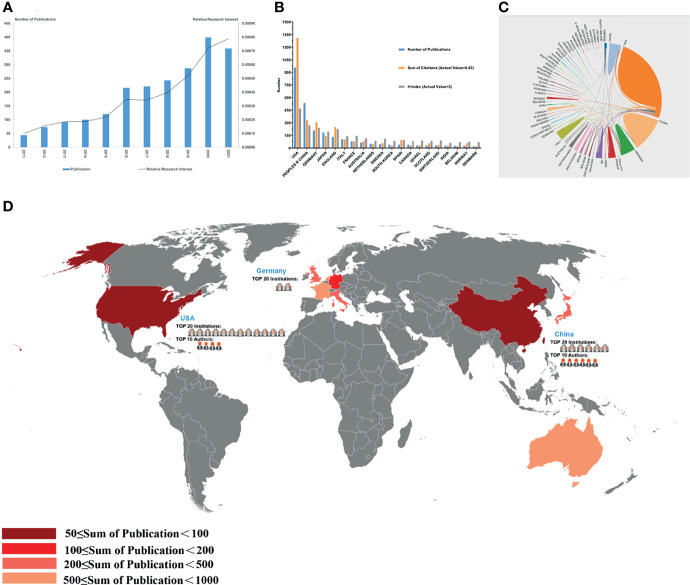
Articles related to the tumor microenvironment of pancreatic cancer published worldwide and by country/region. **(A)** The total publication number and RRI. **(B)** The total publication number, total citations, and H-index of the 20 most productive countries/regions. **(C)** Collaboration between countries/regions based on https://bibliometric.com. **(D)** The publication density map.

The data processing flowchart is shown in **Figure 2**. According to the inclusion criteria, a total of 2,155 papers in the WOSCC database were included (the original data can be found in **Supplement File 2**). The annual publication number increased over time. A total of 70,448 citations and an average of 32.69 citations per paper were noted. The H-index was 109. The number of publications on the TME of PC was highest in 2020 (**Figure 3A**).

### Contribution by Country

A total of 69 countries/regions published studies of the TME of PC. The USA has the greatest number of publications (958 publications, accounting for 44.46% of all publications worldwide; a total of 39,471 citations, with an average of 45.5 citations per paper and an H-index of 94), followed by China (536 publications, accounting for 24.87%, a total of 10,298 citations, with an average of 20.7 citations per paper and an H-index of 54) and Germany (208 publications, accounting for 9.65%; a total of 10,184 citations, with an average of 48.96 citations per paper and an H-index of 48) (**Figure 3B**). The total number of citations of papers from the USA were much higher than those of papers from other countries/regions. Although Germany had fewer publications than China, it had a higher total number of citations, ranking second. Although only 26 papers were published in Norway, the average number of citations of each paper was 72.88, which was the highest among the top 20 countries with the most publications. The USA and Germany cooperated closely with other countries/regions, but China had less cooperation with other countries/regions (**Figure 3C**).

### Contribution by Institution

**Figure 4:**
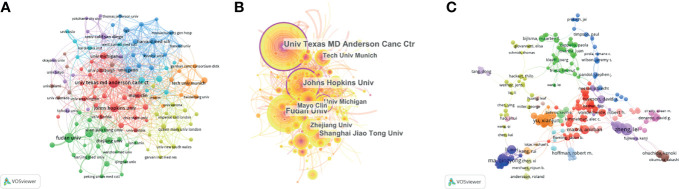
Contributions of institutions and authors to publications on the tumor microenvironment of pancreatic cancer. **(A)** The publication number network of institutions drawn by VOSviewer. **(B)** Network of institutions drawn by CiteSpace. **(C)** Co-authorship cited authors (circle size represents the number of citations). Drawn by VOSviewer.

A total of 2,111 institutions worldwide participated in research in this field. VOSviewer was applied to analyze the institutional citation network. Active institutions were defined as those with no fewer than 10 publications and no fewer than 100 citations. A total of 115 such institutions were identified (**Figures 4A, B**). Among them, the University of Texas M.D. Anderson Cancer Center had the most publications and highest total number of citations. The average citations per paper of the Cold Spring Harbor Laboratory were the highest at 181.60. Of the 20 institutions with the most publications, most were in the USA (12 institutions), followed by China (six institutions) and Germany (two institutions) (**Table 1**).

**Table 1:** The 20 institutes with the most publications on the tumor microenvironment of pancreatic cancer.

Institution	Country	No. of Publications	No. of Citations
UNIV TEXAS MD ANDERSON CANC CTR	USA	96	5025
FUDAN UNIV	PEOPLES R CHINA	79	1446
JOHNS HOPKINS UNIV	USA	66	3694
SHANGHAI JIAO TONG UNIV	PEOPLES R CHINA	52	1110
UNIV MICHIGAN	USA	41	2562
ZHEJIANG UNIV	PEOPLES R CHINA	40	862
TECH UNIV MUNICH	GERMANY	38	1890
UNIV PENN	USA	37	4780
MAYO CLIN	USA	36	901
XI AN JIAO TONG UNIV	PEOPLES R CHINA	36	896
HARVARD MED SCH	USA	35	1189
UNIV CALIF SAN DIEGO	USA	34	1158
GERMAN CANC RES CTR	GERMANY	31	1289
MEM SLOAN KETTERING CANC CTR	USA	31	3005
UNIV NEBRASKA MED CTR	USA	31	2118
NYU	USA	30	2051
NANJING MED UNIV	PEOPLES R CHINA	27	683
CHINESE ACAD MED SCI	PEOPLES R CHINA	26	692
NORTHWESTERN UNIV	USA	26	818
UNIV CALIF LOS ANGELES	USA	26	960

### Author Contributions

A total of 11,933 authors were listed for the 2,155 included papers, and the average number of authors per paper was 5.54. Among the 10 authors with the most publications, six were from China, and four were from the USA (**Table 2**). The most published authors from China were from Shanghai Jiao Tong University. We used VOSviewer to analyze the cocitation network of the authors, and those with more than 50 citations were defined as key researchers. A connection represents cooperation between authors, and the circle size represents the number of citations of an author. David A. Tuveson had the most citations at 4,856 and an average of 107.6 citations per paper (**Figure 4C**).

**Table 2:** The 10 authors who have contributed the most publications on the tumor microenvironment of pancreatic cancer.

Author	Country	Affiliation	No. of Publications	No. of Citations
ZHENG L	PEOPLES R CHINA	Shanghai Jiao Tong University	30	1421
YU XJ	PEOPLES R CHINA	Fudan University	26	431
JAFFEE EM	USA	University of Southern California	25	1752
ZHANG B	PEOPLES R CHINA	Fudan University	21	439
MAITRA A	USA	University of Texas System	20	1330
XU J	PEOPLES R CHINA	Fudan University	18	378
TUVESON DA	USA	Cold Spring Harbor Laboratory	17	4859
DI MAGLIANO MP	USA	University of Michigan	16	1070
LIU C	PEOPLES R CHINA	China Medical University	16	358
SHI S	PEOPLES R CHINA	Fudan University	16	271

### Journals Publishing Research on the TME of PC

A total of 520 journals published papers related to the TME of PC between 2011 and 2021. The data were analyzed using VOSviewer. A total of 102 journals had more than five relevant publications. Among the top 20 journals in terms of the number of publications, Cancer Research had the highest number of publications at 96 (**Table 3**). Nine of these 20 journals were published in the USA, followed by Switzerland (five journals). Cancer Cell had the highest average number of citations for each paper at 271.40, followed by Gut and the Journal of Clinical Investigation. Although Science, Nature Reviews Disease Primers and Nature Cell Biology had average citation numbers per paper of 1,011, 810, and 725, respectively, they were not included in the calculation because they had two or fewer related publications.

**Table 3:** The 20 journals that have published the most articles regarding the tumor microenvironment of pancreatic cancer.

Journal	Country/Region	No. of Publications	No. of Citations
CANCERS	SWITZERLAND	96	756
CANCER RESEARCH	USA	68	4309
CANCER LETTERS	IRELAND	62	1546
ONCOTARGET	USA	61	1820
PLOS ONE	USA	55	1700
SCIENTIFIC REPORTS	ENGLAND	47	943
CLINICAL CANCER RESEARCH	USA	43	2483
PANCREAS	USA	35	580
INTERNATIONAL JOURNAL OF MOLECULAR SCIENCES	SWITZERLAND	34	559
FRONTIERS IN ONCOLOGY	SWITZERLAND	30	154
ONCOIMMUNOLOGY	USA	29	643
FRONTIERS IN IMMUNOLOGY	SWITZERLAND	27	343
ONCOGENE	ENGLAND	27	1378
PANCREATOLOGY	SWITZERLAND	24	410
WORLD JOURNAL OF GASTROENTEROLOGY	USA	22	600
BIOCHIMICA ET BIOPHYSICA ACTA-REVIEWS ON CANCER	NETHERLAND	19	389
CANCER DISCOVERY	USA	18	1584
GASTROENTEROLOGY	USA	18	1521
JOURNAL OF EXPERIMENTAL & CLINICAL CANCER RESEARCH	ENGLAND	18	512
MOLECULAR CANCER	ENGLAND	18	775

### Publication Situation

**Figure 5:**
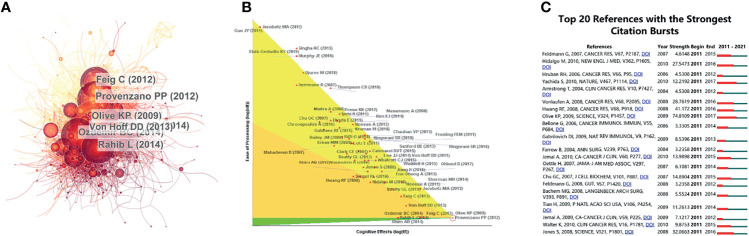
References related to the tumor microenvironment of pancreatic cancer. **(A)** Clustering analysis of the tumor microenvironment of the pancreatic cancer cocitation network drawn by CiteSpace. **(B)** In-depth analysis of the articles with the highest citation rates. **(C)** The 20 references with the strongest citation bursts based on analysis in CiteSpace.

For the study of the TME of PC, among the top 10 most cited publications, three papers were in Cancer Cell (**Table 4**). Cocitations were first proposed as a measure of the relationship between publications by the American informatician scientist Small in 1973, which shows papers with a major impact on a particular field. The included papers cited a total of 5,9561 publications. The top N was set as 50 in CiteSpace software, and a simple cocitation analysis of the publications was conducted (**Figure 5A**). The publications with the greatest impact were further analyzed (**Figure 5B**). Subsequently, the 20 references with the strongest citation bursts (**Figure 5C**) were obtained, which showed that the number of citations per period of a given paper increased rapidly, indicating that the contribution of each paper was relatively significant.

**Table 4:** The 10 most cited articles regarding the tumor microenvironment of pancreatic cancer.

Title	Corresponding authors	Journal	Publication Year	Total Citations
Pancreatic cancer exosomes initiate premetastatic niche formation in the liver	Stanger, BZ	NATURE CELL BIOLOGY	2015	1013
Enzymatic Targeting of the Stroma Ablates Physical Barriers to Treatment of Pancreatic Ductal Adenocarcinoma	Hingorani, SR	CANCER CELL	2012	991
CD40 Agonists Alter Tumor Stroma and Show Efficacy Against Pancreatic Carcinoma in Mice and Humans	Vonderheide, RH	SCIENCE	2011	903
The Pancreas Cancer Microenvironment	Tuveson, DA	CLINICAL CANCER RESEARCH	2012	643
Gemcitabine Plus Bevacizumab Compared With Gemcitabine Plus Placebo in Patients With Advanced Pancreatic Cancer: Phase III Trial of the Cancer and Leukemia Group B (CALGB 80303)	Kindler, HL	JOURNAL OF CLINICAL ONCOLOGY	2010	577
Hyaluronan impairs vascular function and drug delivery in a mouse model of pancreatic cancer	Tuveson, DA	GUT	2013	499
Tumor-Derived Granulocyte-Macrophage Colony-Stimulating Factor Regulates Myeloid Inflammation and T Cell Immunity in Pancreatic Cancer	Vonderheide, RH	CANCER CELL	2012	485
CSF1/CSF1R Blockade Reprograms Tumor-Infiltrating Macrophages and Improves Response to T-cell Checkpoint Immunotherapy in Pancreatic Cancer Models	DeNardo, DG	CANCER RESEARCH	2014	476
Low-Dose Irradiation Programs Macrophage Differentiation to an iNOS(+)/M1 Phenotype that Orchestrates Effective T Cell Immunotherapy	Huber, PE	CANCER CELL	2013	449
Stromal biology and therapy in pancreatic cancer	Tuveson, DA	GUT	2011	445

### Keyword Analysis

**Figure 6:**
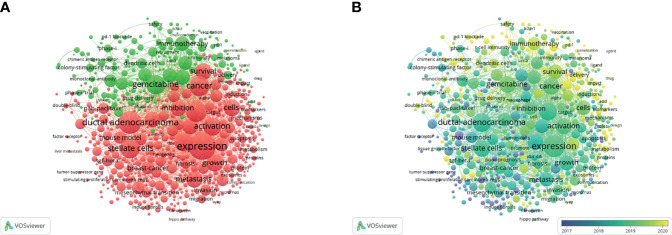
Keywords related to the tumor microenvironment of pancreatic cancer. **(A)** Network visualization of keywords drawn by VOSviewer. **(B)** Overlay visualization of keywords drawn by VOSviewer.

The keyword co-occurrence analysis by VOSviewer visually displayed keywords, which were clustered into two main categories. We summarize these two clusters as basic research (cluster 1, red) and clinical application (cluster 2, green) (**Figures 6A, B**). In cluster 1, the most recent three hot topics were glutamine metabolism (avg. pub. per year as of 2021.300, 23 occurrences), carcinoma-associated fibroblasts (avg. pub. per year as of 2021.174, 36 occurrences) and oxidative phosphorylation (avg. pub. per year as of 2021.167, 13 occurrences). The most recent three hot topics in cluster 2 were liposomes (avg. pub. per year as of 2021.758, 11 occurrences), endoscopic ultrasound (avg. pub. per year as of 2021.534, 9 occurrences) and photodynamic therapy (avg. pub. per year as of 2021.479, 14 occurrences). (**Supplemental File 2**: **Table S1**)

### Dual-Mapping Overlay of the TME of PC

**Figure 7:**
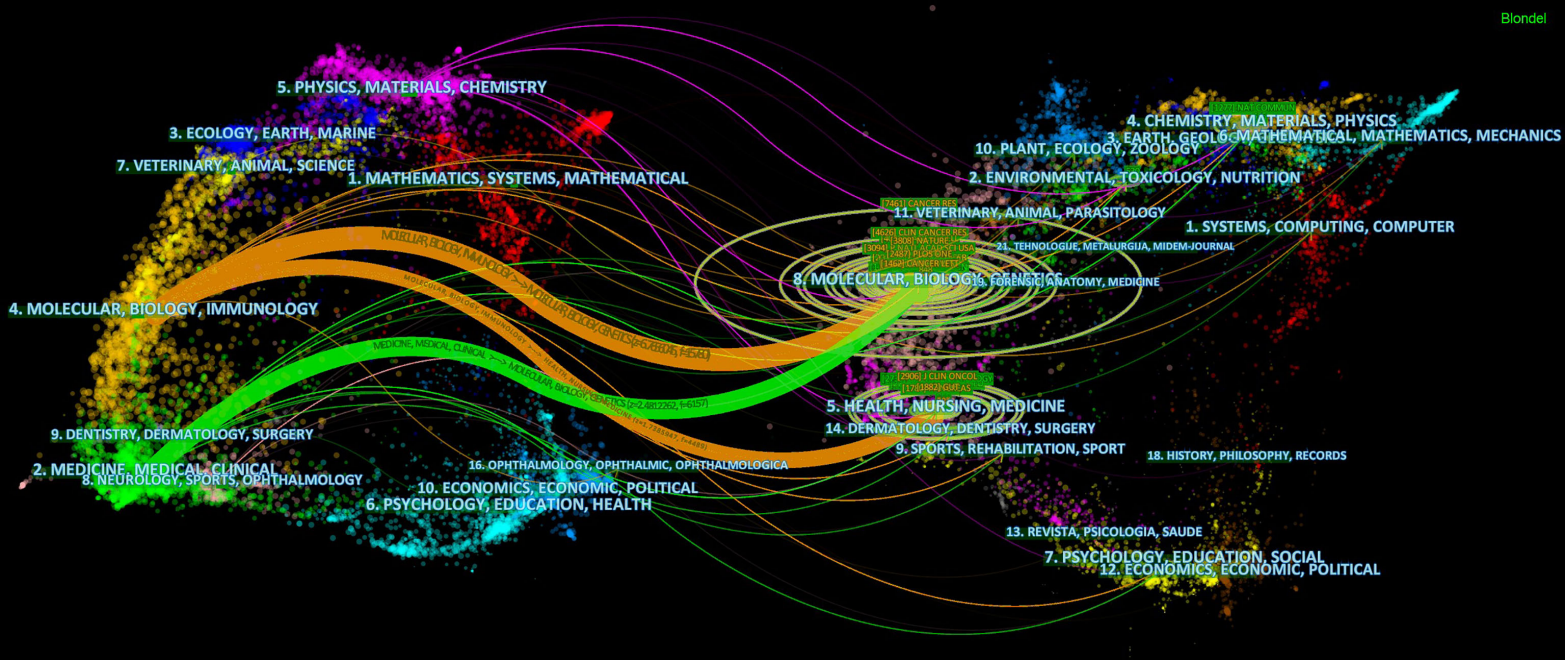
Dual-map overlay with publications on the tumor microenvironment of pancreatic cancer.

The dual-mapping overlay reveals the overall scientific contributions. The left side represents the citing outline, the right side represents the cited outline, and the curve is the quotation association line from the outside to the relevant side. This connection illustrates the flow of knowledge and the relationship between different research areas (**Figure 7**).

### Clinical Trials

Nine completed clinical trials investigated the TME of PC between 2011 and 2021 (**Table 5**), with four from the USA and two each from France and Germany. Among them, four clinical trials mainly evaluated the efficacy and safety of drug combinations with programmed death ligand 1 (PD-L1) antibody; five were related to gemcitabine. Only three clinical trials included more than 40 samples, most of which were in phase I.

**Table 5:** Clinical studies related to the tumor microenvironment of pancreatic cancer between 2011 and 2021.

Study	ClinicalTrials.gov Identifier	Official title	Time	Country	Design	No. of patients	Conditions	Intervention		Phase	Primary purpose	Summary
								Treatment group	Comparison group			
1	NCT03331562	A SU2C Catalyst^®^ Trial of a PD1 Inhibitor With or Without a Vitamin D Analog for the Maintenance of Pancreatic Cancer	2017.12.27-2020.06.29	Germany	Randomized, Parallel Assignment, Quadruple (Participant, Care Provider, Investigator, Outcomes Assessor)	24	Pancreatic Cancer, Pancreas Adenocarcinoma, Advanced Pancreatic Cancer, Metastatic Pancreatic Cancer, Metastatic Pancreatic Adenocarcinoma	Drug: Pembrolizumab, Drug: paricalcitol	Drug: placebo	Phase 2	Treatment	The study is being conducted to determine the effects that pembrolizumab, with or without the addition of paricalcitol, may have on pancreatic cancer. Half of the patients will be randomized to receive pembrolizumab + paricalcitol and half to receive pembrolizumab + placebo.
2	NCT03168139	Olaptesed (NOX-A12) Alone and in Combination With Pembrolizumab in Colorectal and Pancreatic Cancer	2017.04.18-2020.03.25	Germany	N/A, Single Group Assignment, None (Open Label)	20	Metastatic Colorectal Cancer, Metastatic Pancreatic Cancer	Drug: Olaptesed pegol - Monotherapy, Drug: Olaptesed pegol + Pembrolizumab - Combination Therapy	NO	Phase 1	Treatment	The purpose of this study is to show that the type, number and/or distribution of tumor metastases infiltrating immune cells such as cytotoxic T cells and/or the cytokine signature in the tumor metastases can be modulated by treatment with olaptesed pegol and to explore safety, tolerability and efficacy of olaptesed pegol in combination with pembrolizumab as a basis for subsequent studies in combination with immunotherapies, in particular checkpoint inhibitors.
										Phase 2		
3	NCT02777710	Evaluation of Safety and Activity of an Anti-PDL1 Antibody (DURVALUMAB) Combined With CSF-1R TKI (PEXIDARTINIB) in Patients With Metastatic/Advanced Pancreatic or Colorectal Cancers	2016.06-2019.11	France	N/A, Single Group Assignment, None (Open Label)	48	Colorectal Cancer, Pancreatic Cancer, Metastatic Cancer, Advanced Cancer	Drug: Pexidartinib, Drug: Durvalumab	NO	Phase 1	Treatment	The study is to conduct a Phase I dose escalation study in order to evaluate the safety and clinical activity of a combined treatment associating an anti-CSF1R (PEXIDARTINIB) with an anti-PD-L1 (DURVALUMAB) in patients with advanced/metastatic colorectal or pancreatic cancers. Dose escalation part will determine the Maximum tolerated dose (MTD) and recommended phase 2 dose (RP2D) of Pexidartinib given in combination with Durvalumab. Extension part will evaluate the clinical activity of the combination at the RP2D.
4	NCT02179970	To Assess the Safety of Continuous IV Administration of Plerixafor in Patients With Advanced Pancreatic, Ovarian and Colorectal Cancers	2015.06-2018.12.14	France	N/A, Single Group Assignment, None (Open Label)	26	Pancreatic Adenocarcinoma Metastatic, Ovarian Serous Adenocarcinoma, Colorectal Cancer Metastatic	Drug: Plerixafor	NO	Phase 1	Treatment	The purpose of this study is to find out if the study drug has the same effect on patients with advanced pancreatic, ovarian or colorectal cancer, as they have seen in our laboratory experiments, and find out the right dose of the study drug to give.
5	NCT02030860	A Randomized Pilot/Pharmacodynamic/Genomic Study of Neoadjuvant Paricalcitol to Target the Microenvironment in Resectable Pancreatic Cancer	2014.01-2015.12	USA	Randomized, Parallel Assignment, None (Open Label)	15	Adenocarcinoma of the Pancreas	Drug: Paricalcitol, Drug: Abraxane, Drug: Gemcitabine	NO	Not Applicable	Treatment	This study is a randomized pilot/pharmacodynamic/genomic study of neoadjuvant paricalcitol to target the microenvironment in resectable pancreatic cancer to determine the effect of targeting the vitamin D metabolic program in the tumors of patients treated with one cycle of gemcitabine/abraxane with or without paricalcitol prior to surgery for resectable pancreatic cancer through an assessment of cellular and imaging markers.
6	NCT01989000	The Role of the Tumor Microenvironment of Pancreatic Cancer to Predict Treatment Outcome	2013.11-2017.12	Netherlands	Nonrandomized, Parallel Assignment, None (Open Label)	47	Pancreatic Cancer	Drug: Gadobutrol, Drug: [F-18]HX4, Drug: Gemcitabine, Radiation: Radiotherapy, Procedure: Pancreaticoduodenectomy	NO	Not Applicable	Diagnostic	The study is to use tumor cellularity and extracellular matrix composition to assess non-invasively *in vivo* by diffusion weighted magnetic resonance imaging (DWI) and tumor vascularity can be assessed by dynamic contrast enhanced magnetic resonance imaging (DCE-MRI). Tumor hypoxia can be evaluated by T2* MRI and PET-CT, using the 18F-labeled hypoxic marker HX4.
7	NCT01903083	Chemoimmunotherapy and Radiation in Pancreatic Cancer	2013.07-2017.12	USA	N/A, Single Group Assignment, None (Open Label)	10	Locally Advanced Malignant Neoplasm	Drug: Tadalafil, Drug: Gemcitabine, Radiation: Radiation, Procedure: Pancreaticoduodenectomy	NO	Phase 1	Treatment	The goal of this study is to evaluate the safety of combination treatment that includes chemotherapy, radiation therapy, and immunotherapy in patients with pancreatic cancer.
8	NCT01715142	Effect on Tumor Perfusion of a Chemotherapy Combining Gemcitabine and Nab-paclitaxel (Abraxane) in Pancreatic Cancer	2013.03.21-2015.09.21	USA	N/A, Single Group Assignment, None (Open Label)	23	Pancreatic Adenocarcinoma Resectable, Pancreatic Adenocarcinoma Locally Advanced, Pancreatic Adenocarcinoma Metastatic.	Drug: Gemcitabine, Drug: Abraxane	NO	Early Phase 1	Treatment	This proof-of-concept trial is studying the “dynamic” tumor response after the administration of a short course of gemcitabine and nab-paclitaxel (Abraxane) (a) during a window interval (4 weeks = 1 cycle) before surgery in resectable pancreatic cancer (cohort 1 = 21 patients) and (b) during at least 8 weeks (2 cycles) in locally advanced or metastatic pancreatic cancer (cohort 2 = 10 patients).
9	NCT02546531	Defactinib Combined With Pembrolizumab and Gemcitabine in Patients With Advanced Cancer	2015.09-2021.05	USA	Nonrandomized, Parallel Assignment, None (Open Label)	43	Pancreatic Cancer	Drug: Gemcitabine Drug: Defactinib Drug: Pembrolizumab	NO	Phase 1	Treatment	Focal adhesion kinase (FAK) inhibitors have demonstrated reasonable anti-tumor activity in the preclinical setting. A maximal synergetic effect was achieved when a FAK inhibitor was given in combination with a PD-1 antagonist and chemotherapy in multiple pancreas tumor animal models. This supports the concept of using FAK inhibitors to reduce stromal fibrosis during checkpoint immunotherapeutic treatment. Therefore, these robust preclinical findings will be tested in the proposed phase I trial.

## Discussion

In this study, we used VOSviewer and CiteSpace software to conduct a visualization analysis of 2,155 papers related to the TME of PC published between 2011 and 2021 to help researchers understand the current situation of global publications and predict future research hotspots. From this study, the publication number and RRI for the TME of PC increased significantly, indicating that the popularity of research in this field has gradually increased (**Figure 3A**). Developed countries accounted for most of the top 20 countries/regions in terms of publications and cooperation with other countries/regions (**Figure 3C**). The H-index of the USA was the highest, and the same trends were observed in studies on PD-1 and PD-L1 in the field of cancer (20, 21). Twelve institutions in the USA appeared in the top 20 institutions (**Table 1**). Of the 10 publications with the most citations, three were from US scholar David A. Tuveson, who made outstanding contributions to the establishment of *in vitro* organ models of PC, metastasis mechanisms, immunotherapy, and drug delivery (22) (**Table 4**), which indicates that the economic strength of the country is closely related to its scientific research (**Figure 3D**). As a developing country, China has experienced rapid growth in the number of publications in this field, from only four publications in 2011. Since 2015, the number of annual publications and the total number of publications in China have exceeded those of Germany, becoming the country with the second most publications. In the field of cancer research, the number of publications in China has increased significantly in recent years (23, 24), probably because cancer has become a major cause of death in China since 2010, and China has increased its investment in scientific research on public health and other fields in recent years (25). Notably, the total citations and the average number of citations of papers published by Chinese scholars were relatively low, and none of the 10 most cited papers were published by Chinese scholars, indicating that the quality of papers published by Chinese researchers needs to be further improved. In addition, China has shown limited cooperation with other countries/regions on the topic of the TME of PC, and its maturity in this field is still relatively limited, which also explains the low H-index in China.

The CiteSpace literature cocitation map showed that the paper published by Provenzano (2012) (26) has had a very important impact in this field (**Figures 5A, B**). Hyaluronic acid (HA) in the extracellular matrix (ECM) is the main stromal determinant of high interstitial fluid pressures in pancreatic ductal adenocarcinoma (PDAC) and fibrosis. Provenzano et al. showed that an enzymatic agent can dissolve interstitial HA, and combined with gemcitabine, it can permanently reshape the TME. This study elucidated the principle of action of HA in the ECM of PC cells in detail and provided a new direction for the clinical treatment of PDAC. Olive (2009) (27), Rhim (2014) (28), Ozdemir (2014) (29), and Rahib (2014) (30) made outstanding contributions to exosomes, ECM, and immunotherapy in PC and provided new insight for later studies.

Keywords represent the main topic of publications. Through the co-citation analysis of VOSviewer, it was found that the whole body of research can be divided into two clusters: the basic research cluster and the clinical research cluster. In basic research, the latest three keywords are “glutamine metabolism”, “carcinoma-associated fibroblasts”, and “oxidative phosphorylation”, which is also one of the future research hotspots predicted by this study. Reviewing and summarizing related studies, we found that these three keywords mainly focus on the direction of tumor energy metabolism and malignant biological behavior changes in the hypoxic microenvironment (31). Tumor hypoxia is mainly caused by defects in the vascular system in rapidly growing solid tumor tissue. Coupled with the limitation of the local space, the supply of oxygen and nutrients is reduced, and it is easy to form a hypoxic microenvironment in the local tumor (32). Although its growth can be inhibited to a certain extent through energy metabolism, for some malignant solid tumors, hypoxia can stimulate tumor cell metabolism and changes in malignant behavior, providing a suitable environment for the survival of pancreatic cancer cells (33). Studies have shown that the hypoxic microenvironment is a key factor in tumor progression, immune escape, and treatment tolerance (34, 35).

**Figure 8:**
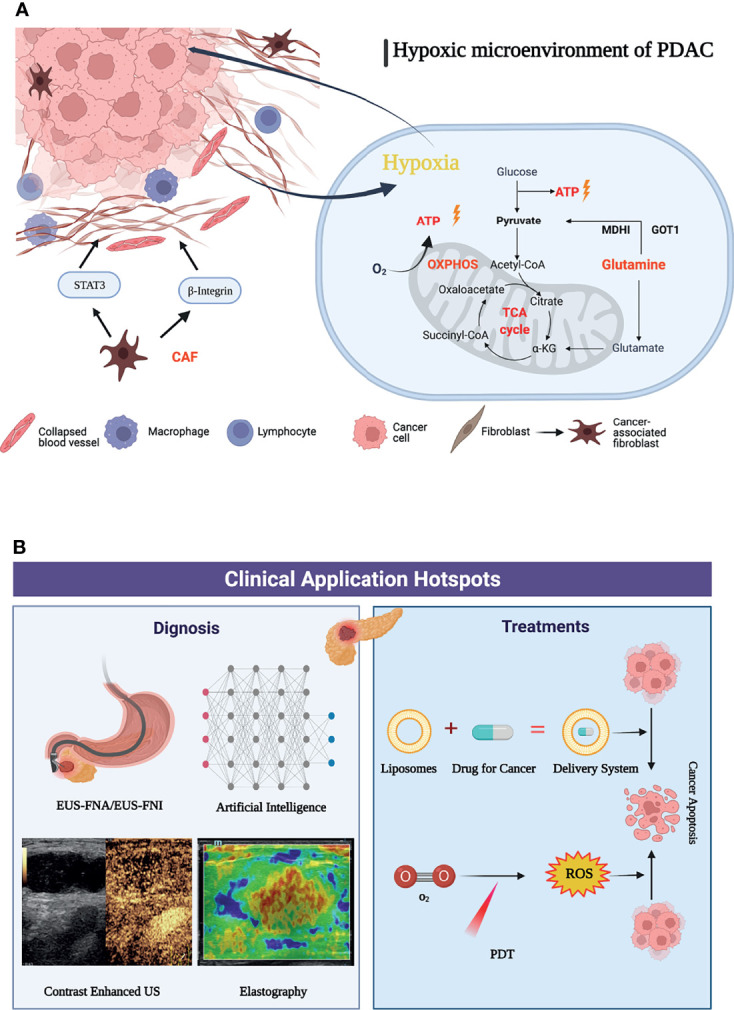
Graphic abstract of current research hotspots of pancreatic cancer tumor microenvironment. **(A)** Mechanisms associated with a graphic abstract of the hypoxic microenvironment in PDAC. **(B)** The hotspots in clinical application of pancreatic cancer tumor microenvironment.

Pancreatic cancer is rich in interstitial cells and abundant extracellular matrix, lacks vascularization, and exhibits higher levels of hypoxia than most solid tumors (36). The hypoxic tumor microenvironment has a wide range of effects on the biological behavior or malignant phenotype of pancreatic cancer, including invasion and metastasis as well as pathological angiogenesis, synergistically promoting the development and treatment resistance of pancreatic cancer, which has been recognized as an independent prognostic factor (37, 38). During solid tumor development, cancer cells are often embedded in “cancer cell nests” surrounded by stromal cells, especially cancer-associated fibroblasts (CAFs). Importantly, cancer-associated fibroblasts (CAFs) can modify the metabolism of adjacent cancer cells, so that its activity can promote tumor growth, invasion and angiogenesis. CAFs can deposit and remodel the ECM and increase β1 integrin and signal transducer and activator of transcription 3 (STAT3) signal transduction, which leads to fibrosis and increased tissue tension, further causing vascular dysfunction and hypoxic tumor microenvironment. To adapt to the hypoxic microenvironment, PDAC cells also enhance the utilization of the reprogramming of amino acid glutamine metabolism as a carbon fuel source for ATP (39). Glutamine, one of the most abundant amino acids in blood, plays a variety of important roles in the survival and proliferation of cancer cells and has become an important intermediate for cancer cells involved in TCA in the process of infinite proliferation (40). PDAC cells can also exhibit “non-canonical” glutamine metabolism, characterized by utilization of the glutamine catabolic enzyme GOT1 and maleate dehydrogenase 1 (MDH1) to generate pyruvate that is the main source of NADPH for PDAC cells to maintain fatty acid synthesis (41, 42). In addition, the hypoxic microenvironment can also lead to an increase in oxidative phosphorylation. Overexpression of key mitochondrial components, such as UQCRC1 (mitochondrial ubiquinol-cytochrome c reductase core protein 1), leads to increased mitochondrial oxidative phosphorylation (OXPHOS) and ATP production, thereby maintaining the rapid proliferation rate of PDAC cells (43). Oxidative phosphorylation also promotes the stemness and immune evasion properties of pancreatic cancer stem cells (44). Based on this, the metabolic reprogramming mechanism in the hypoxic microenvironment of pancreatic cancer has been continuously explored (**Figure 8A**). The research and application of targeted adjuvant therapy has attracted increasing attention from researchers (38). Studies have shown that glutaminolysis can be controlled by glutaminase succinylation, leading to the inhibition of tumor growth (45, 46). Coadministration of a glutaminase inhibitor with gemcitabine also abrogated MUC5AC (Mucin 5AC)-mediated gemcitabine resistance in mouse and human pancreatic cancers, providing a new target for future immunotherapy (47). In addition, studies have shown that mTORC1 and other substances can affect glutamine synthetase to inhibit the synthesis of glutamine, further inhibit the nutrient uptake of pancreatic cancer cells, and reduce the proliferation ability of PDAC (48). At present, DX3-235, DX3-213B and other targeted oxidative phosphorylation inhibitors can effectively inhibit the growth of pancreatic cancer cells in the low nanomolar range and have no obvious toxicity (49). Therefore, it was believed that targeting the hypoxic microenvironment of PDAC, employing a multimodal therapeutic approach and developing biointegration targets, and remodeling the TME are promising strategies.

When using cluster analysis for clinical application by VOSviewer, it was found that there were relatively few clinical research studies. This shows that the focus of researchers has gradually shifted from basic experiments to clinical applications with a primary concentration on the diagnosis, identification and adjuvant treatment of pancreatic cancer, the efficient delivery of drugs and the reduction of side effects, and the development and application of novel therapies. “Endoscopic ultrasound”, “liposomes”, and “photodynamic therapy” were the highly concerned research topics of clinical applications in recently. Among the keywords of clinical application, endoscopic ultrasound (EUS) has surpassed CT and PET-CT and has gradually become an important method for the diagnosis and prognosis of pancreatic cancer. It has received extensive clinical attention due to its unique advantages of real-time dynamic observation and no radiation. The application of new imaging methods, such as contrast material-enhanced ultrasonography and elastography, can accurately identify pancreatic tissue and necrotic areas through imaging modalities, such as the hardness of the lesion and blood flow distribution, and can effectively identify benign and malignant pancreatic masses (50). The application of artificial intelligence has also improved the performance of EUS, which can assist doctors in making more accurate diagnoses (51). EUS-FNA (endoscopic ultrasound fine-needle aspiration) has become a first-line method for the diagnosis of pancreatic cancer due to its high accuracy and few complications, with a sensitivity of over 90% (52, 53). Biomarkers such as proteins and mRNAs enriched in EUS-FNA-derived fluid can be used to evaluate the expression of genes in the tumor microenvironment, providing the possibility of early diagnosis of pancreatic cancer (54). With the development of technology, the application of EUS in adjuvant therapy has gradually attracted increasing attention. As a method of antitumor drug delivery, EUS-FNI (endoscopic ultrasound guided fine needle injection) not only injects drugs into the lesion to improve their effectiveness but also implants medical markers to realize targeted radiotherapy (55–57). EUS-guided brachytherapy combined with targeted drugs such as gemcitabine-based 5-fluorouracil is also expected to increase the proportion of pancreatic cancer patients who convert to resectable disease and provide more durable local control than conventional therapy (58).

In terms of clinical treatment, reducing the resistance of chemotherapeutic drugs, enabling drugs to penetrate fibrotic tumor tissues and precisely delivering them to tumor cells have become hot topics for researchers (59). Liposomes gradually attracted attention as novel drug carriers in the pancreatic cancer tumor microenvironment after 2015. Nanoliposomal irinotecan (NAL-IRI) has been developed as a preparation to improve drug delivery and reduce side effects. Recent data suggest that early intervention with nal-IRI in the treatment modality significantly improves median OS and PFS in pancreatic cancer. Onivyde (intravenous liposomal irinotecan injection) is approved for use in combination with 5-fluorouracil and leucovorin (5-FU/LV) in patients with metastatic pancreatic adenocarcinoma that has progressed following gemcitabine-based therapy and was developed to overcome the pharmacological and clinical limitations of nonliposomal irinotecan (60–62). Vactosertib (a selective small-molecule inhibitor of the TGF-β type I receptor kinase ALK5) in combination with paclitaxel constructs nanoliposome complexes to increase drug solubilization while overcoming the barriers to drug penetration by PDAC matrix utilization and reducing side effects (63). Liposome-encapsulated collagenase type-I can also disassemble the dense PDAC collagen stroma, destroy and reshape the tumor microenvironment, and increase drug penetration into the pancreatic tumor.

At present, dynamic therapy (such as photodynamic therapy, sonodynamic therapy, and photothermal therapy) has become an important part of research on adjuvant therapy of pancreatic cancer (64). PDT (photodynamic therapy) relies on light-activated photosensitizers and reactive oxygen species produced by oxygen to kill tumor cells while promoting an antitumor response of the immune system (65). Moreover, PDT can effectively improve the fibrotic tumor microenvironment (66). PDT combined with chemotherapy under the guidance of ultrasound/CT can significantly promote drug penetration and reduce drug resistance to targeted drugs (67). In recent years, with the advancement of nanotechnology, the development of new photosensitizers and the application of new photosensitive liposomes, recombinant exosomes and other nanodrug carriers can solve the problems of the limitation of photodynamic therapy in irradiation depths and hypoxic environments to a certain extent (68). For example, multi-inhibitor-loaded nanoliposomes combined with photodynamic therapy can be co-delivered in tumors, while spatiotemporal control of drug release can also reduce systemic drug exposure and related toxicity, which provides a new prospect for the precise treatment of cancer (69). In the future, under the premise of achieving accurate diagnosis of pancreatic cancer through endoscopic ultrasound, the construction of new nanodrug delivery systems, such as liposomes, combined with new therapies (such as photodynamic therapy), is expected to further improve the survival rate of pancreatic cancer (**Figure 8B**).

The dual-map overlay shows that the studies on the TME of PC mainly focus on the fields of medicine and biology (**Figure 7**). However, as research progresses, various disciplines have gradually interpenetrated each other. For example, Matai et al. constructed a tumor-specific microenvironment for PC by a three-dimensional bioprinting technique (70), and Zhao et al. used a drug delivery system based on nanocarriers to accelerate the death of immunogenic cells in PC (71). Multidisciplinary integration allows more comprehensive research, resource sharing, and the benefits of complementary advantages, which in turn deepens research levels.

The current poor efficacy of multiple radiochemotherapies and immunotherapies is closely related to the TME of PC. One of the main reasons may be the highly immunosuppressive characteristics of the TME and stromal components (72). Therapies targeting immune checkpoints in the TME of PC have been considered very important (73). However, the nine current clinical trials on the TME of PC are mostly single-center clinical trials, with small numbers of patients and a low level of evidence, and most of them are phase I trials. These shortcomings may be due to the complexity of the TME of PC (74), the interaction between fibroblasts and tumor-associated macrophages, and the fact that substances such as HA provide scaffolds for cytokines and growth factors, which directly promote tumor cell growth (75). All these factors form an immunosuppressive environment that leads to drug resistance (76). Therefore, therapies that consider both the combination of the cells and stroma of the TME and the combination of antitumor drugs hold promise. For example, the clinical trial NCT03168139 mainly focuses on one of the important targets, C-X-C motif chemokine 12 (CXCL12), which prevents the entry of immune cells into the PC region. NOX-A12 (olaptesed pegol) targets the destruction of CXCL12 to allow activated T cells to enter tumors and kill them (77). NOX-A12 combined with a PD-1 inhibitor was used to treat recurrent and refractory PC according to the Response Evaluation Criteria in Solid Tumors. In 25% of patients, the condition was stable, and 70% of the patients survived until 24 weeks, confirming that the combined use of NOX-A12 and PD-1 inhibitors can stabilize the condition of patients with recurrent and refractory PC and improve survival time. However, the sample size of this study was relatively small, rendering it prone to selection bias. Further evaluation of the toxic side effects and safety of drugs is needed.

### Limitations

There are several limitations that should be considered. First, using bibliometrics only allows for a very rough measurement of the research progress. Second, we only used the WOS database to collect literature for analysis. For more comprehensive results, databases such as Scopus or Google Scholar could be adopted in further studies. Third, we conducted a bibliometric study from 2011 to 2021. Some important and landmark studies may have been omitted. In addition, some hotspots that researchers are working on that have not been proven feasible may be missed, resulting in some new hotspots not being included. Fourth, apart from ranking country, institution, author and journal, only VOSviewer and CiteSpace were used for visualization and scientific analysis, the more scientific and common statistical methods like regression analysis will be explore and applied on the bibliometric research, hoping to have unexpected gains in the future. However, the current literature-based bibliometric studies undoubtedly lay the foundation for scholars to quickly understand the research hotspots and development trends of the tumor microenvironment of pancreatic cancer.

## Conclusion

Pancreatic cancer is a severe disease with high morbidity and mortality. Moreover, the mechanism of the TME has extensive application prospects. To sum up, bibliometrics was used to summarize and analyze global research trends on the TME of PC. The trend of the overall publications shows annual increases, and interest in this research is increasing. The USA has the most publications in this field, and China’s publications are increasing at the fastest rate, which enlighten that it is necessary for countries to strengthen academic cooperation to help research progress. Glutamine metabolism, carcinoma-associated fibroblasts, oxidative phosphorylation, liposomes, endoscopic ultrasound and photodynamic therapy have been research hotspots in this field in recent years, which indicated that the current research hotspots mainly focus on energy metabolism in the hypoxic tumor microenvironment, cancer associated fibroblasts in regulating the tumor microenvironment, accurate diagnosis, drug delivery and new treatments.

## Data Availability Statement

The original contributions presented in the study are included in the article/**Supplementary Material**. Further inquiries can be directed to the corresponding authors.

## Author contributions

DX and XS conceived and designed the study. KW, YL, and LL prepared the manuscript and contributed equally to the study. YP and HP prepared the tables and figures. DX, XS, YP and HP reviewed and revised the manuscript. All authors contributed to the article and approved the submitted version.

## Funding

This work was supported by the Foundation of Science and Technology, Department of Sichuan Province (2020YJ0485).

## Conflict of Interest

The authors declare that the research was conducted in the absence of any commercial or financial relationships that could be construed as a potential conflict of interest.

## Publisher’s Note

All claims expressed in this article are solely those of the authors and do not necessarily represent those of their affiliated organizations, or those of the publisher, the editors and the reviewers. Any product that may be evaluated in this article, or claim that may be made by its manufacturer, is not guaranteed or endorsed by the publisher.

## Supplementary Material

Supplemental File 1 Raw data. Detailed information on 2,155 articles was obtained from the Web of Science (this database is publicly available).

Supplemental File 2
**Table S1**. Keywords for the tumor microenvironment in pancreatic cancer.

## Abbreviations

TCGA, The Cancer Genome Atlas.

## Appendix

Search strategies: The search strategy was as follows: [TS=(Pancreatic Neoplasms OR Neoplasm, Pancreatic OR Pancreatic Neoplasm OR Pancreas Neoplasms OR Neoplasm, Pancreas OR Neoplasms, Pancreas OR Pancreas Neoplasm OR Neoplasms, Pancreatic OR Cancer of Pancreas OR Pancreas Cancers OR Pancreas Cancer OR Cancer, Pancreas OR Cancers, Pancreas OR Pancreatic Cancer OR Cancer, Pancreatic OR Cancers, Pancreatic OR Pancreatic Cancers OR Cancer of the Pancreas OR Pancreatic Ductal Adenocarcinoma OR PDAC)] and [TS=(Tumor Microenvironment OR Microenvironment, Tumor OR Microenvironments, Tumor OR Tumor Microenvironments OR Cancer Microenvironment OR Cancer Microenvironments OR Microenvironment, Cancer OR Microenvironments, Cancer)].
